# Experimental evaluation of the impact of household aerosolized insecticides on pyrethroid resistant *Aedes aegypti*

**DOI:** 10.1038/s41598-018-30968-8

**Published:** 2018-08-22

**Authors:** Lyndsey Gray, Sergio Dzib Florez, Anuar Medina Barreiro, José Vadillo-Sánchez, Gabriela González-Olvera, Audrey Lenhart, Pablo Manrique-Saide, Gonzalo M. Vazquez-Prokopec

**Affiliations:** 1 Department of Epidemiology, Emory University, Atlanta, GA USA; 2 Unidad Colaborativa de Bioensayos Entomológicos, Campus de Ciencias. Biológicas y Agropecuarias, Universidad Autónoma de Yucatán, Mérida, Yucatán, Mexico; 3 Entomology Branch, Division of Parasitic Diseases and Malaria, Center for Global Health, Centers for Disease Control and Prevention, Atlanta, GA USA; 4 Department of Environmental Sciences, Emory University, Atlanta, GA USA

## Abstract

The extensive reliance on insecticides to control *Aedes aegypti* mosquitoes and disrupt transmission of dengue, chikungunya and Zika has fueled the emergence of widespread resistance to insecticides. Mismatch between the frequency of pyrethroid resistance in mosquitoes and the occurrence of pyrethroid-based insecticide applications for vector control is often hypothesized to be due to household use of commercial insecticide products. We experimentally quantified phenotypic and genotypic responses of four *Ae*. *aegypti* strains (three field, pyrethroid resistant, and one laboratory, pyrethroid susceptible) after exposure to two commonly used household aerosol insecticide products (a space spray and a residual spray formulation) containing pyrethroid active ingredients. Experiments were performed within homes of Mérida, Mexico. After exposure to the products, all three pyrethroid resistant field *Ae*. *aegypti* strains had significantly lower mortality rates (averaging 41% and 50% for the two products, respectively) than the controls (99%). Applying insecticides as surface sprays led to a significant increase in the frequency of I1016 *kdr* homozygotes in surviving *Ae*. *aegypti*, suggesting strong selection pressure for this allele. Given the large-scale use of household aerosol insecticide products in areas that are endemic for *Ae*. *aegypti*–transmitted diseases, their role as a pyrethroid resistance selection source, particularly when used as space sprays, should be taken into consideration when designing resistance management plans.

## Introduction

Control of insect pests has long relied on pesticide-based interventions. It is not surprising that, as the intensity and geographic spread of chemical control increases, insects have developed genetic, enzymatic and behavioral mechanisms to overcome the toxic effects of insecticides^1,2^. There is well-documented evidence of evolution of resistance to the four major insecticide classes used in public health interventions (pyrethroids, carbamates, organophosphates and organochlorines), with resistance to pyrethroids dominating in insect vectors of major human and animal diseases^3^. Since their development in the 1970s, pyrethroids have been the most widespread chemicals employed for the control of insect vectors due to their low cost, low mammalian toxicity, and high insecticidal capability^1^.

Pyrethroids are classified into two groups based on their chemical structure: type II pyrethroids have a *cyano* moiety at the α-position, while type I pyrethroids do not^4^. This difference results in distinctive toxicological effects on target organisms^1^ and affects the residual capacity of a given insecticide. Resistance to both groups of pyrethroids has been extensively documented in insects. Single nucleotide polymorphisms (SNPs) in genes encoding the voltage-gated sodium channel, also known as *knock-down resistance* (*kdr*) mutations, are associated with pyrethroid resistance in arthropods^5^. When *kdr* mutations are located within or near receptor sites, they can prevent optimal binding of pyrethroids to the sodium channel^5–7^. This disrupts the action potentials needed for normal electrical and chemical signaling, leading to death by paralysis^1,5,8–10^.

Continued pyrethroid exposure in *Anopheles spp*. mosquitoes due to the scale-up of long-lasting insecticide treated bednets is seen as a major driver of pyrethroid resistance in malaria vectors^11–14^. Similarly, ultra-low volume (ULV) applications of insecticides have led to rapid increases in pyrethroid resistance in urban *Aedes* *aegypti* mosquitoes^15,16^. While such examples are well acknowledged and backed with scientific evidence, in specific settings there may be additional selection sources. For example, pyrethroid resistance in malaria vectors can be compounded by the use of the same insecticide class in agriculture^17,18^. Likewise, in northern Argentina and Bolivia, resistance to pyrethroids in the Chagas disease vector *Triatoma infestans* was linked to both vector control and the indoor use of the same insecticides by villagers who had originally obtained the insecticides for agricultural purposes^19^. In Boa Vista, Brazil, the risk of resistance in *Ae*. *aegypti* adults in zones that received intensive deltamethrin ULV spraying in response to a dengue outbreak was similar to the risk in a zone that did not receive the deltamethrin intervention^20^. The intensification of vector control measures in Boa Vista alone did not account for the dramatic increase in pyrethroid resistance status, prompting the authors to hypothesize that household insecticides may play a significant role in resistance selection^20^. Particularly for *Ae*. *aegypti*, the use of consumer-based aerosolized insecticides represents an unmeasured but potentially significant source of pyrethroid resistance evolution. Surveys from the city of Mérida, Mexico, show that 87% of households have and regularly use pyrethroid-based commercial insecticide products as a way to respond to high mosquito numbers^21,22^. However, no study has yet quantified the contribution of such pyrethroid-based products as selection sources of resistance.

A randomized controlled trial conducted in Mérida, Mexico, showed that high levels of deltamethrin resistance in local *Ae*. *aegypti* populations can render deltamethrin-based interventions ineffective^23^. The present study follows up on that trial by experimentally investigating the role of commercial aerosolized insecticides as selection sources for pyrethroid resistance in *Ae*. *aegypti*. As vector control programs replace pyrethroids with other insecticides to which local mosquitoes remain susceptible (e.g., carbamates, organophosphates), a key, unanswered question remains: can pyrethroid susceptibility be regained if such insecticides are rotated out of current vector control interventions? To answer this question, the role of insecticide resistance selection sources that fall outside the purview of programmatic vector control must be quantified. Thus, our experiments were designed to quantify the effect of spraying commercial aerosolized insecticides on the frequency of the I1016 allele and deltamethrin resistant phenotypes in both pyrethroid-resistant and susceptible *Ae*. *aegypti*. Simultaneously, we sought to evaluate how the mode of application (space versus surface spraying) affected survival among pyrethroid-resistant and susceptible *Ae*. *aegypti* strains.

## Results

### Field surveys

Commercial insecticides are not only commonly found in Mérida households, but are also used often and frequently against insect pests. Nearly 94% of households reported using commercial insecticides on a regular basis (Table 1). This percentage may actually under-quantify total insecticide use, since half of all households reported using more than one type of insecticide regularly (Table 1). Participants that applied insecticide regularly reported that they mostly used aerosols (87%), bought commercial insecticides on average 2.9 (±3.0 SD) times over the prior 3 months, applied insecticide 1–3 times a day (63%), and preferred to spray insecticide in the air (46%) or over specific household surfaces (53%). Among those who reported using aerosols, formulations containing the pyrethroids tetramethrin, phenothrin and allethrin dominated as space sprays (51%) (marketed as targeting primarily flying insects, including mosquitoes) and formulations containing the pyrethroids cypermethrin, cyfluthrin and imiprothrin dominated as residual surface sprays (38%) (marketed as targeting ants, scorpions, and cockroaches) (Table 1). For the subsequent space spray and surface spray experiments, we selected the commercial product containing tetramethrin, allethrin, and phenothrin (Raid® House and Garden, coded as ‘space spray formulation’) and the commercial product containing cypermethrin and imiprothrin (Baygon® Multi-Insect Killer, coded as ‘residual spray formulation’).

**Table 1:** Survey results regarding commercial household insecticide use within 150 households in three suburbs of the city of Mérida, Mexico.

Characteristic	N^o^ Houses	Total (%) or Mean (SD)
Brand of insecticide (any kind) most used (n = 148)		
None used	9	6.1
Killer	10	6.8
Raid	53	35.8
Baygon	62	41.9
H24	12	8.1
Ortho	2	1.4
Type of insecticide most commonly used (n = 141)		
Aerosol	122	86.5
Plug-in	9	6.4
Coil	9	6.4
Other	1	0.7
Active ingredients of most common aerosolized insecticides (n = 109)		
Tetramethrin, allethrin, and phenothrin	59	54.1
Cypermethrin and imiprothrin	24	21.2
Cyfluthrin and imiprothrin	9	17.0
Cyfluthrin	5	9.4
Tetramethrin and phenothrin	5	9.4
Permethrin, proxopur, and prallethrin	4	3.7
Tetramethrin, proxopur, and fenvalerate	2	1.8
Unidentified	1	0.9
Other additional insecticides used regularly? (n = 141)		
Yes	71	50.4
Secondary insecticides commonly used (n = 66)		
Aerosol	24	17.8
Plug-in	18	13.3
Coil	18	13.3
Other	6	4.4
Number of times purchased (within the past 3 months)	135	2.9 (3.0)
Average use (times per day) (n = 141)		
Not used every day	45	31.9
1–3	89	63.1
4–6	6	4.3
7–9	1	0.7
10+	0	0.0
Means of application in the home (n = 122)		
Applied as a space spray	56	45.9
Applied as a surface spray	64	52.4
Applied directly to mosquitoes	2	1.6

### Space Spray Trials

**Figure 1:**
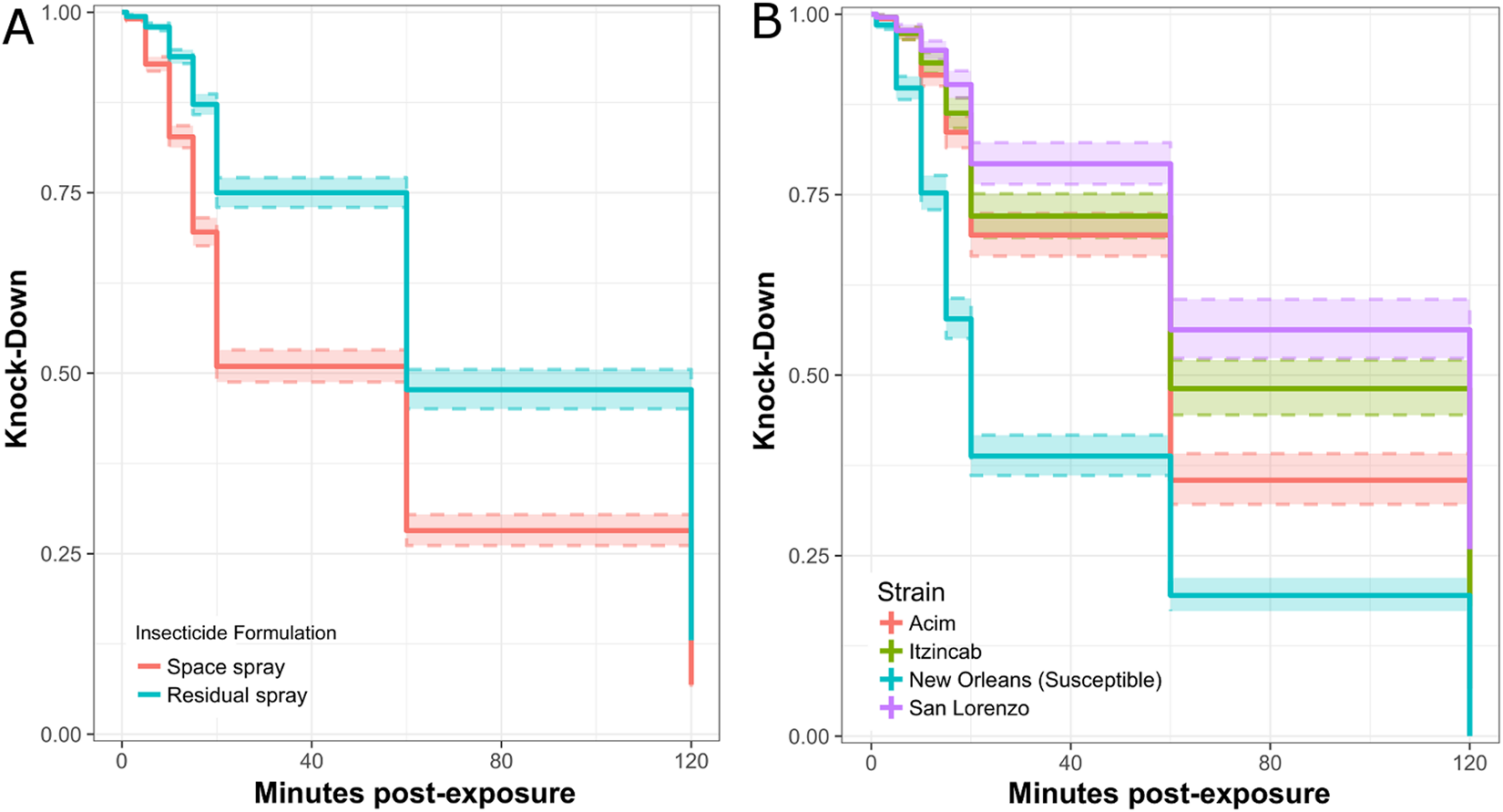
*Ae*. *aegypti* knock-down in space spray trials, stratified by insecticide (**A**) and mosquito colony (**B**). Colored bands indicate 95% CI for knock-down estimate.

Applying insecticide according to its correct manufacturer instructions significantly affected knock-down rates; the space spray formulation caused more rapid knock-down than the residual surface spray formulation (log-rank [LR] test: 234.9, p < 0.0001) (Fig. 1A). As expected, significant differences in knock-down were also observed between the mosquito strains, with higher knock-down observed in the susceptible strain compared to the three resistant field strains (LR test: 668.9, p < 0.0001) (Fig. 1B).

The mean percent mortality only varied by mosquito strain (New Orleans: 100%, Acim: 63%, Itzincab: 44%, San Lorenzo: 43%) (overall F-test: 6.69, p < 0.001). Interestingly, the mode of insecticide application had no significant effect on mortality (space spray formulation: 52%, residual surface spray formulation: 56%) (overall F-test: 0.12, p = 0.73). A post-hoc Tukey test for the two-way ANOVA showed significant difference in mean percent mortality only for the susceptible vs. resistant field strain comparisons (F-test: 4.82, p < 0.05) but not for any of the resistant field strains with each other (all p-values > 0.05). As such, we aggregated all colonies for further mortality estimates.

**Figure 2:**
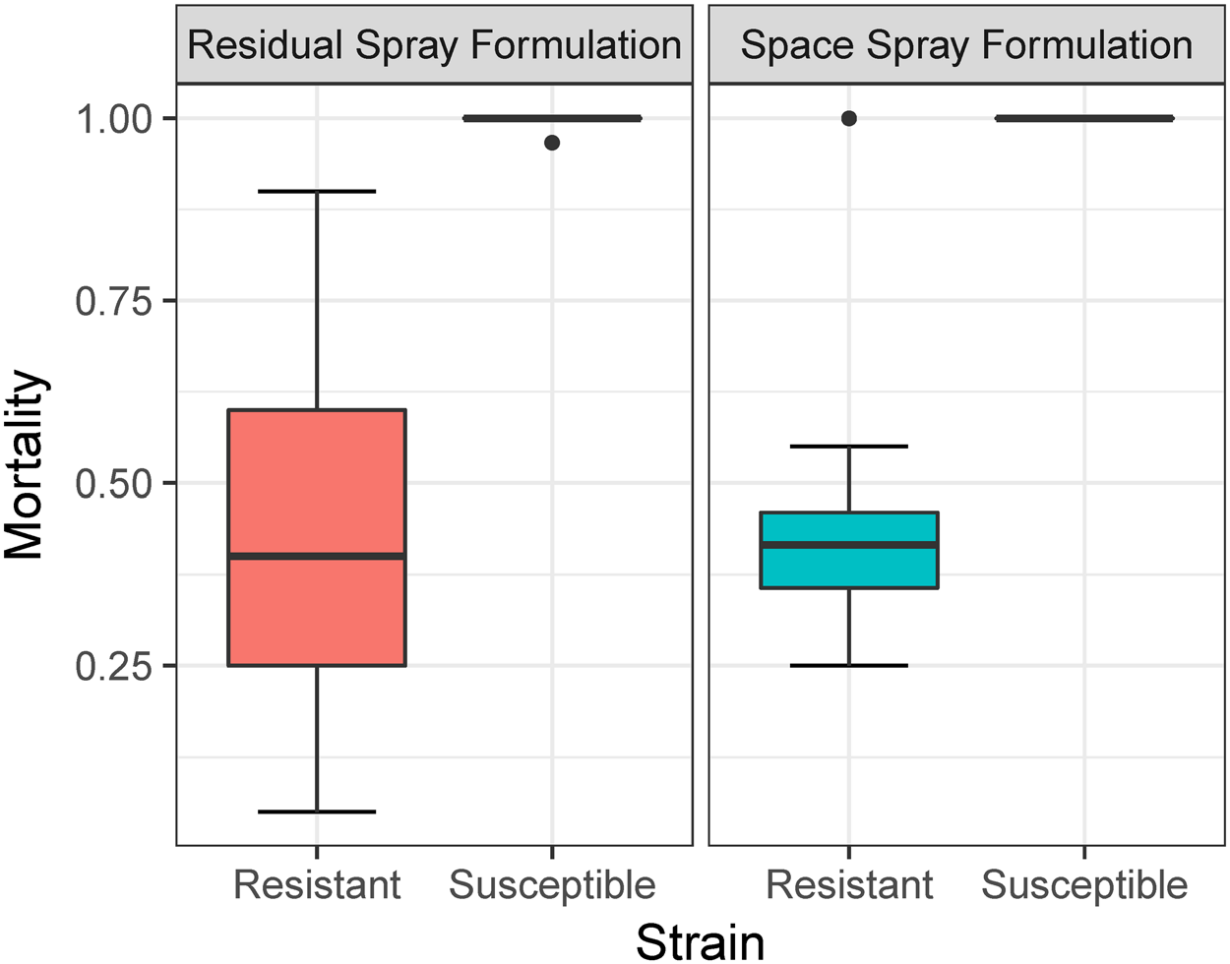
*Ae*. *aegypti* relative median mortality for space spray trials, stratified by insecticide and resistance status of tested mosquito strains. Dots point to two outlier observations.

**Figure 3:**
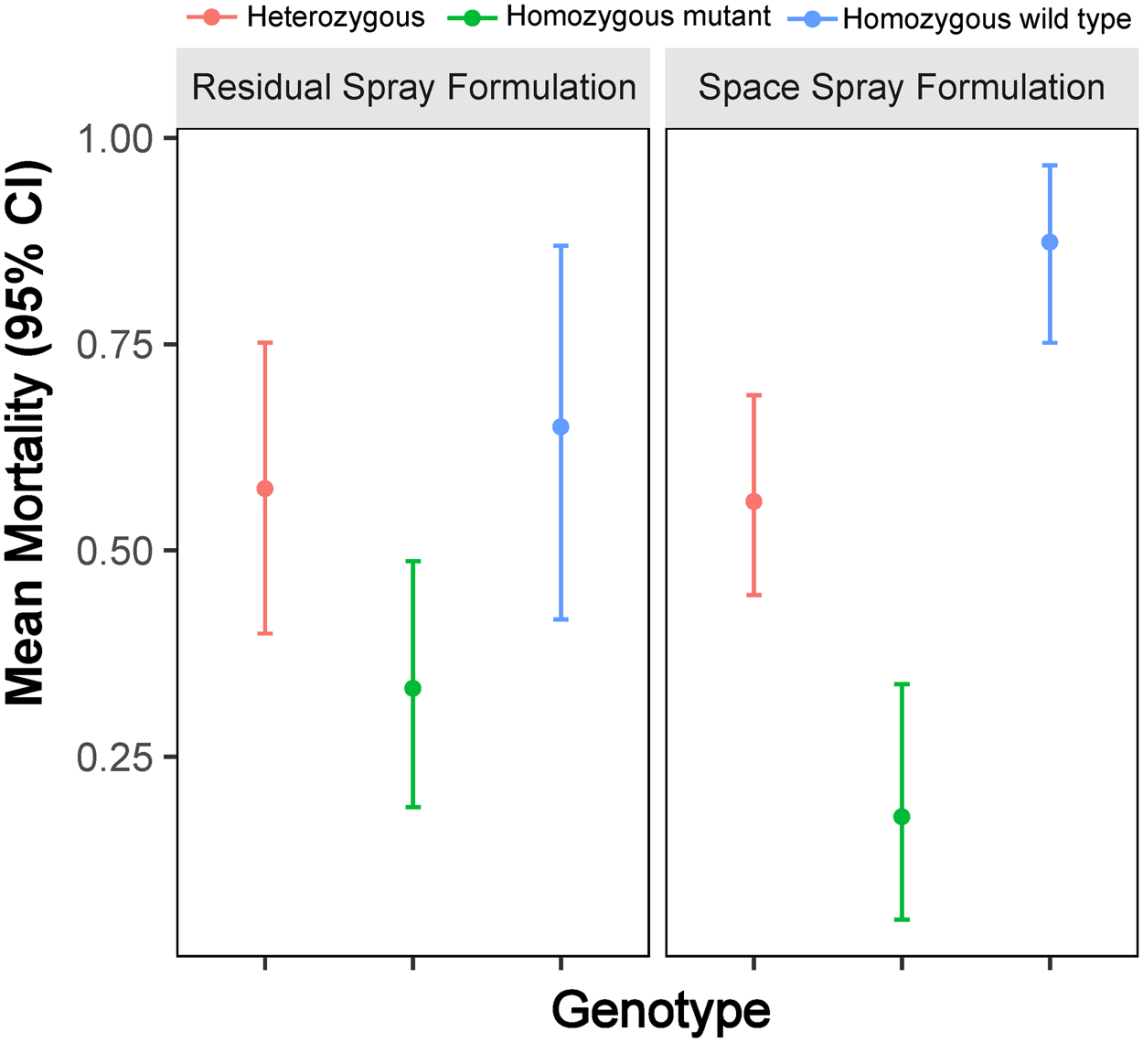
Mean relative mortality for different I1016 genotypes exposed to two pyrethroid insecticide formulations in space spray trials.

Overall median mortality was 44% (inter-quartile range, IQR: 20–83%) for the space spray formulation and 41% (IQR: 18–72%) for the residual spray formulation (Fig. 2). A GLMM including insecticide and mosquito strain as predictor variables showed a significant and decreased odds of mortality for the field resistant *Ae*. *aegypti* strains (Table 2). Interestingly, the presence of the I1016 *kdr* mutation was associated with a significant reduction in the odds of mortality, which was significantly higher for the homozygous resistant mosquitoes compared to the homozygous wild type (Table 1). Significantly higher isoleucine allele frequency was observed among survivors than non-survivors for all three resistant field strains (Acim: 76% vs. 52%, Itzincab: 75% vs. 57%, San Lorenzo: 80% vs. 48%) (Table 3). Individuals that were homozygous for I1016 showed the lowest mean mortality (10–40%) compared to homozygous wild type (V1016) individuals (91–100%) (Fig. 3).

**Table 2:** Parameter estimates of a GLMM quantifying the association between *Ae*. *aegypti* mortality (response variable) and resistance variables for space spray trials.

Characteristic	OR	95% CI (OR)
Resistance colony^a^		
Acim	0.34	0.19, 0.61
Itzincab	0.11	0.04, 0.37
San Lorenzo	0.04	0.01, 0.23
New Orleans	1.00	—
Resistance allele frequency^b^		
Heterozygous (V/I)	0.25	0.18, 0.34
Homozygous mutant (I/I)	0.06	0.03, 0.12
Homozygous wild type (V/V)	1.00	—

^a^Parameter and OR estimates derived from a GLMM equation with resistance colony and insecticide as covariates.

^b^Parameter and OR estimates derived from a GLMM equation with resistance colony, insecticide, and genotype as covariates. Resistance alleles for the I1016 *kdr* mutation.

**Table 3:** I1016 genotypes and phenotypes for *Ae*. *aegypti* tested across four replicates of space spray trials.

Resistance Colony	Survival Status	I1016 Genotype			Total (n)	P-Value^a^	Allele Frequency for I	95% CI^b^
		V/V	V/I	I/I				
Acim	Died	19	43	22	84	0.0002	0.52	0.41, 0.62
	Survived	5	17	34	56		0.76	0.64, 0.87
	Total	24	60	56	140		0.61	0.53, 0.69
Itzincab	Died	11	28	19	58	0.0071	0.57	0.44, 0.70
	Survived	6	30	49	85		0.75	0.66, 0.84
	Total	17	58	68	143		0.68	0.60, 0.75
San Lorenzo	Died	13	27	11	51	<0.0001	0.48	0.34, 0.62
	Survived	4	27	56	87		0.80	0.71, 0.88
	Total	17	54	67	138		0.68	0.60, 0.76

^a^P-values measure significant association between I1016 genotype and survival status among each resistant colony.

^b^95% confidence intervals are calculated for allele frequency for I.

### Surface Spray Trials

**Figure 4:**
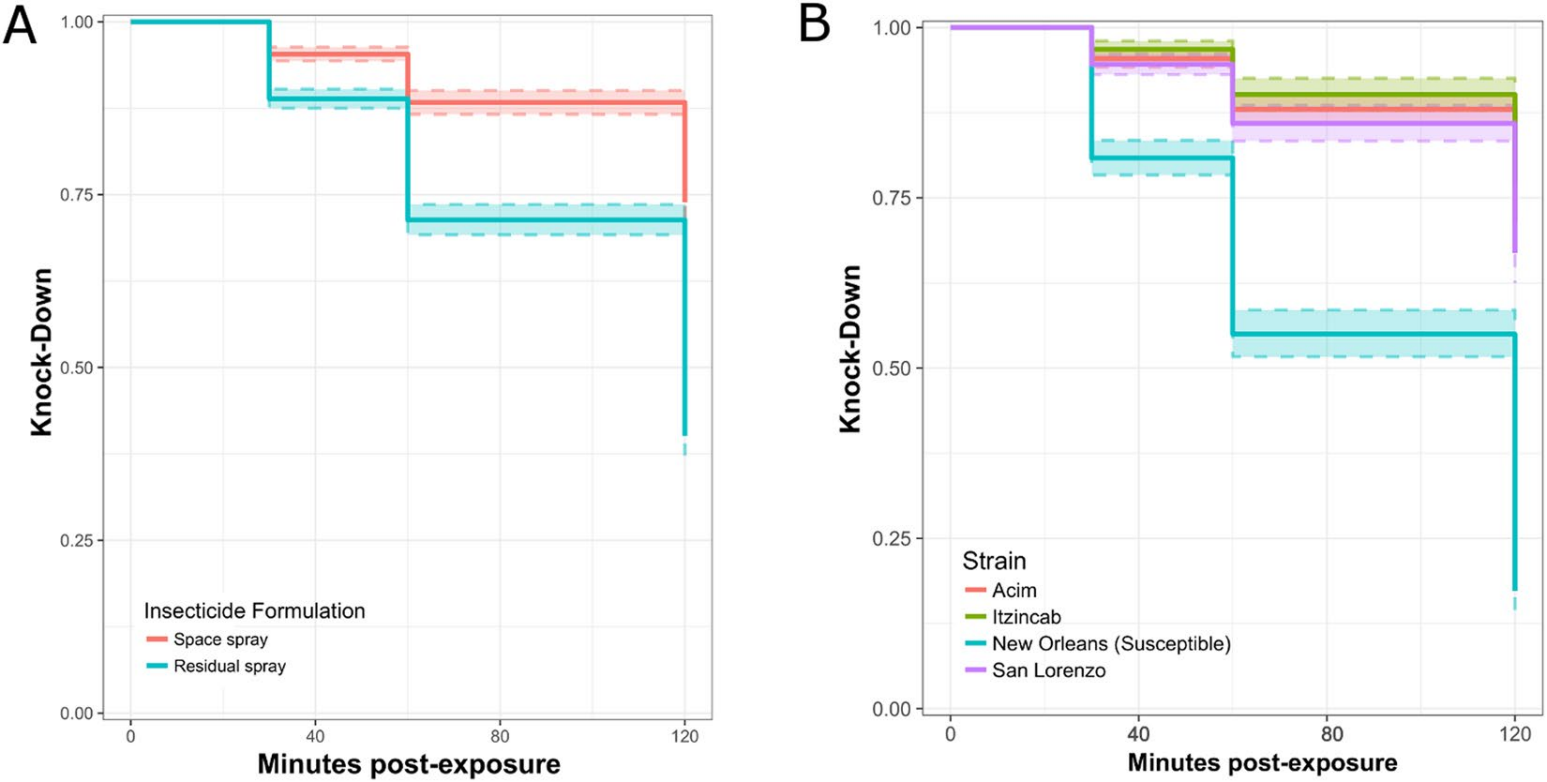
*Ae*. *aegypti* knock-down in the surface spray trials across all six days, stratified by insecticide (**A**) and mosquito colony (**B**). Colored bands indicate 95% CI for knock-down estimate.

When both insecticides were applied as surface sprays, higher mosquito knock-down was observed when insecticide was applied according to its recommended mode of application. In comparison to the space spray formulation, the residual surface spray formulation resulted in greater knock-down compared to the space spray formulation (LR test: 229.5, p < 0.0001) (Fig. 4) (Table S1). That difference was most pronounced on the initial day of insecticide application and declined steadily across the remaining three days (Fig. 4) (Table S1). A significant difference in knock-down was also observed between mosquito strains (LR test: 695.7, p < 0.0001) (Fig. 4). The difference in mean percent knock-down overall was only significant between the susceptible strain and each of the three resistant field strains (Post-hoc Tukey tests; p-values < 0.05) (Fig. 4). Significance in knock-down rates among the four mosquito strains was maintained across all four days (Table S1).

**Figure 5:**
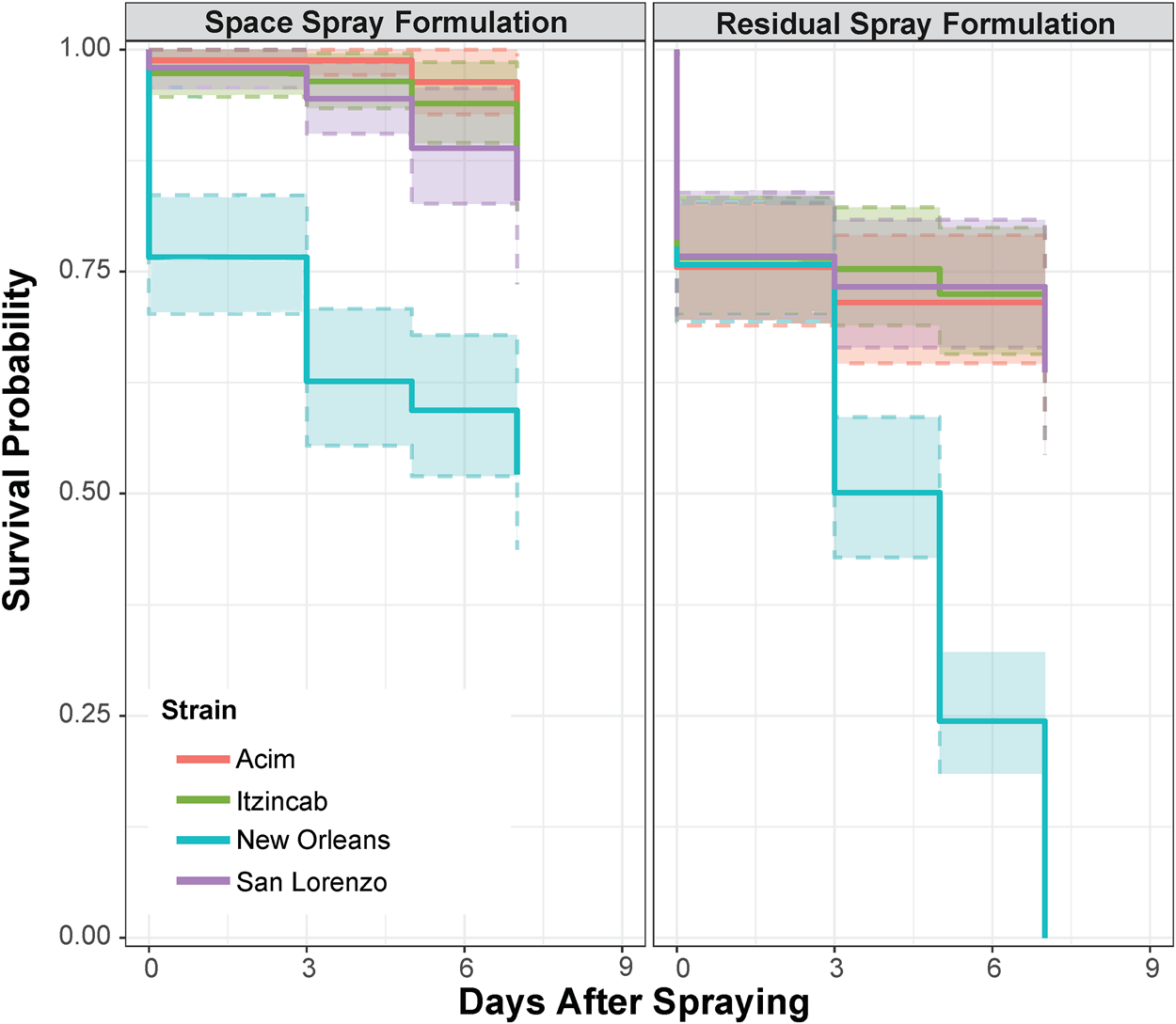
*Ae*. *aegypti* mortality for surface spray trials, stratified by insecticide and mosquito colony. Dashed line colored bands indicate 95% CI for survival estimate.

Predictably, the residual surface spray formulation caused greater cumulative mortality (323/616 mosquitoes, 48%) than the space spray formulation (94/616, 15%) when applied on walls. Overall mortality (aggregating data from four days of exposure) was relatively low among the three resistant field strains, with mortality in all resistant field strains being significantly lower than in the susceptible strain (LR test > 242.0, p < 0.0001) (Fig. 5). Cumulatively, half of the mosquitoes from the New Orleans susceptible strain died (235/471, 50.1%), whereas only 10% of mosquitoes from Acim (52/477), 12% of mosquitoes from Itzincab (57/470), and 13% of mosquitoes from San Lorenzo (57/454) died. No significant differences were observed in allele frequency among survivors and non-survivors (p-values for all three colonies >0.05, Table 4).

**Table 4:** I1016 genotypes and phenotypes for *Ae*. *aegypti* tested across eight replicates of surface spray trials.

Resistance Colony	Survival Status	I1016 Genotype			Total (n)	P-Value^a^	Allele Frequency for I	95% CI^b^
		V/V	V/I	I/I				
Acim^c^	Died	3	17	24	44	0.0557	0.74	0.61, 0.87
	Survived	19	53	39	111		0.59	0.50, 0.68
	Total	22	70	63	155		0.63	0.56, 0.71
Itzincab^c^	Died	4	13	32	49	0.3981	0.79	0.67, 0.90
	Survived	10	39	56	105		0.72	0.63, 0.81
	Total	14	52	88	154		0.74	0.67, 0.81
San Lorenzo^c^	Died	8	14	22	44	0.183	0.66	0.52, 0.80
	Survived	7	33	54	94		0.75	0.66, 0.84
	Total	15	47	76	138		0.72	0.65, 0.80

^a^P-values measure significant association between I1016 genotype and survival status among each resistant colony.

^b^95% confidence intervals are calculated for allele frequency for I.

^c^Only mosquitoes from Acim, Itzincab, and San Lorenzo colonies tested on the initial day of insecticide exposure and six days post-exposure were analyzed via RT-PCR.

## Discussion

Our results provide empirical evidence of the significant negative impact of pyrethoid resistance on the efficacy of commercial household aerosolized insecticides. Furthermore, our findings suggest that such consumer products (particularly when applied as space sprays) may be an additional selection source for pyrethroid resistance in *Ae*. *aegypti*. Exposing three pyrethroid-resistant field strains to two commercial aerosolized insecticides with different active ingredients led to significantly reduced mortality in field pyrethroid resistant *Ae*. *aegypti* strains compared to the susceptible strain. In our experiments, only half of the mosquitoes in resistant populations survived exposure to commercial aerosolized insecticides and, in space spray trials, frequency of *kdr* I1016 homozygotes significantly increased as a consequence of such exposure. Our findings are of particular relevance for the design of insecticide resistance management plans, and confirm the need to account for the influence of household use of aerosolized insecticides if pyrethroid resistance is to be restored.

As experiments were performed in actual homes and used each product’s recommended mode of application, our estimates quantify the differential efficacy of commercial products in the context of field resistant *Ae*. *aegypti* populations. A recent study evaluating thirteen commercial aerosolized products commonly sold in Mexico showed high variability in *Ae*. *aegypti* mortality (41–100%) due to insecticide type and formulation and mosquito location within a room^24^. Unfortunately, that study did not test the products against pyrethroid-susceptible and resistant strains, limiting the quantification of loss of efficacy due to resistance. Our results showed that pyrethroid resistance leads to a reduction in space spray efficacy of ~50% for both space and surface spray formulations. Given the significant investment by householders in commercial aerosolized insecticides^25^, understanding and addressing the loss of product efficacy on *Ae*. *aegypti* due to resistance can lead to improved formulations and modes of application. This, in turn, can help increase the value of this form of community-based vector control. For instance, the addition of synergists (e.g., piperonyl butoxide) to existing pyrethroid formulations may help increase insecticide efficacy if resistance is mediated by metabolic mechanisms^26^.

Although many commercial aerosolized insecticides are marketed as targeting *Ae*. *aegypti* (or ‘Zika vectors’), they are actually formulated to target a wide range of insects. Changes in formulations that include new chemical groups can become a concern, particularly if those same chemical groups are also approved for public health use in vector control. In Mexico, *Ae*. *aegypti* are fully susceptible to carbamates^27,28^, making it an important insecticide class for urban vector control. Household commercial products with formulations including the carbamate propoxur are now available in Mexico. Such propoxur-based products are sold as surface sprays. Our study indicates that surface spray pyrethroid formulations lead to low mortality of pyrethroid-resistant field mosquitoes and (when used as space sprays) to a high frequency of survivors that are I1016 *kdr* homozygotes. Should a similar effect hold for carbamates, this will mean that the role of these new formulations in changing the resistance landscape of *Ae*. *aegypti* populations cannot be discounted. The shift away from pyrethroids in vector control is driven by their low efficacy against field resistant populations^23^. Given the marked fitness costs to *kdr*^29^, the hope is that this shift will help restore pyrethroid susceptibility. The heavy use of pyrethroid-based products by the community, combined with the findings from our experiments, emphasizes a key component that deserves more attention: can pyrethroid susceptibility be restored in the context of high household pyrethroid insecticide use, or is it better to guard other insecticide classes for which *Ae*. *aegypti* is still susceptible (e.g., carbamates) by limiting their adoption within commercial products? Addressing such question will not only require more research (particularly selection experiments looking at the impact of insecticides over generations and in the field) but also a more fluent communication between commercial insecticide producers and vector control authorities. Such connection is critical if true *Ae*. *aegypti* insecticide resistance management is to be achieved.

Low mortality rates among resistant populations of *Ae*. *aegypti* (as observed in^23^) may cause people to underestimate or discredit the protective effects of mosquito control programs. A recent study in Ecuador found that, when distrust in local vector control interventions was coupled with increases in mosquito-transmitted disease, families invested in household insecticide products^25^. This could also help explain why our survey results indicated that the vast majority of surveyed households regularly used commercial aerosolized insecticides. However, the decision to purchase a particular mosquito control product does not appear to be based on guidance or interventions provided by vector control authorities. Instead, they are often based solely on perceived product effectiveness and cost^25,30^. Within Mérida alone, the median annual estimated expenditure per household for all products used to kill insect pests was 408 Mexican pesos (approximately US$31). This suggests an annual market for commercial insecticides of over 75 million Mexican pesos (>US$5.7 million)^31^. Given the important role commercial aerosolized insecticides may play as community-based tools for *Ae*. *aegypti* control, they must be considered as a key component of urban integrated vector management and taken into consideration when designing insecticide resistance management plans.

Survivor phenotypes among pyrethroid-resistant *Ae*. *aegypti* may be explained through multiple *kdr* mutations as well as other potential mechanisms of resistance^32^. Previous studies^27,33^ and analyses of a random sample of 100 adult female *Ae*. *aegypti* from this study (50 survivors and 50 dead from the space spraying trial) showed that another *kdr* mutation (C1534) was found in ~100% of all tested mosquitoes. Mosquitoes with a double mutant I1016/C1534 haplotype exhibit a higher degree of pyrethroid resistance than those with a V1016/C1534 haplotype^34–36^, providing a justification for the low efficacy of aerosolized insecticides against field resistant strains, in comparison to the control strains. Other resistance mechanisms, which we were unable to quantify, such as increased metabolic activity, may also contribute towards pyrethroid resistance and should be analyzed in future studies^3,37,38^. Given that commercial aerosolized insecticide formulations contain a wide array of pyrethroids (e.g., Table 1 and^24^), assessing the specificity of resistance mechanisms responsible for observed phenotypes will require quantifying the role of each molecule in selecting for specific resistance mechanisms. In addition, as this study did not explicitly quantify selection, future studies should focus on the evolutionary consequences of repeated insecticide applications over multiple generations and on various pyrethroid resistance mechanisms, both in the field and the lab. Recent evidence shows that environmental conditions (e.g., larval habitats^33^) or individual level variation (e.g., attributes of the gut microbiota^39^) can greatly affect mosquito resistant phenotypes. Consequently, assessing the evolutionary implications of household insecticide use will require a comprehensive study integrating laboratory and field experimentation. Such information, supported by the initial results provided by our study, can provide the ultimate response to the hypothesis suggesting that household insecticides are a strong selection pressure for pyrethroid resistance.

## Methods

### Study Design

In the first stage of this study, we performed household surveys to identify community behaviors regarding the use of consumer-based aerosolized insecticides as well as to identify specific pyrethroid-based commercial products to use in our experiments. We determined: a) which active ingredients were present in the most frequently used commercial insecticide products, b) how often, on average, commercial insecticide products were used on a daily basis, and c) what were the common modes of application for each commercial product.

Surveys were conducted in three neighborhoods of the city of Mérida, Mexico, in June 2016. Mérida is the capital of the Yucatán State and is the largest and most populous (population ~1 million) city in the Yucatán Peninsula. Mérida is also consistently one of the districts in Mexico with the highest number of dengue cases^31^. Additionally, recent studies indicate that householders in this area have a high rate of usage of consumer-based aerosolized insecticides^21^, and that local *Ae*. *aegypti* populations have high levels of pyrethroid resistance^23,27^. In total, 150 households were randomly selected from three neighborhoods that had participated in a randomized controlled trial evaluating the impact of pyrethroid resistance on indoor residual spraying interventions^23^.

Survey results informed the second phase of the study: experimental trials that evaluated how exposure of *Ae*. *aegypti* to commercial insecticide products commonly used by householders impacted mosquito knock-down and survival. As similar insecticide formulations exist for multiple brand name commercial products, we selected the top two formulations (in terms of reported frequency and quantity used) irrespective of their brand name or manufacturer.

Experiments were performed in laboratory and semi-field settings in Mérida and involved a factorial design, which included the two top insecticide formulations, two modes of application (space spray or residual surface spray) and four *Ae*. *aegypti* strains. By allowing for the interaction between insecticide formulation and mode of application, our study also quantified the selection pressure of insecticides when used in accordance with manufacturer guidelines or not.

*Ae*. *aegypti* eggs were collected from the three survey neighborhoods: Acim, Itzincab, and San Lorenzo, which were known to have high levels of pyrethroid resistance^23^. CDC bottle bioassays performed prior to the experiments on F1 female *Ae aegypti* characterized all three strains as highly resistant, with average 24-hour mortalities ranging from 12% (Itzincab) to 54% (San Lorenzo) (Fig. S1). The susceptible *Ae*. *aegypti* New Orleans laboratory strain was used as a control. From the three resistant field strains of *Ae*. *aegypti*, only sugar-fed, F1- or F2-generation females between 2–5 day-old were used in experiments. For the New Orleans strain, only sugar-fed females between 2–5 days old were used.

Space spray experiments involved adapting previously cited protocols to quantify mosquito knock-down and mortality^40,41^. Cylindrical, nylon, mesh bioassay cages (approximately 25 cm × 18 cm diameter) were hung from a stand, each containing 25 *Ae*. *aegypti* from one of the four strains (Fig. S2A). Fifteen minutes prior to insecticide exposure, the stands and four cages were placed in a sealed room (5.1 m. × 5.1 m × 2.7 m) with no air conditioning or air circulation. Before applying the insecticide, temperature and humidity measurements were recorded using a digital thermo-hygrometer (Extech 44550). Insecticide was applied by a technician wearing appropriate personal protective equipment (gloves and mask) at an upwards 45° angle, one meter away from the bioassay cages (Fig. S2A). Insecticide was sprayed from left to right for ten seconds exactly (using a digital timer). This procedure was followed for the space spray formulation for a total of 4 replicates, and then repeated using the residual spray formulation for other 4 replicates. All cages were washed with detergent and all metal frames were rinsed with acetone between each replicate and trial. For each replicate, knock-down was quantified at 1, 5, 10, 15 and 20 minutes after spraying. After 20 minutes, all mosquitoes were aspirated from the mesh cages, placed in polystyrene foam cups covered in mesh netting, and provided cotton soaked in 10% sucrose. Two other observations for knock-down were made at 60 and 120 minutes after spraying. Mortality was then assessed for all mosquitoes 24 hours post-spray.

For the surface spray experiment, we modified the World Health Organization’s standardized protocols for residual spraying of mosquito adulticides. Specifically, we used two uninhabited houses with no recent (>1 year) history of insecticide use located in the Umán neighborhood of Mérida. Both houses were identical in building materials (cement walls) and floorplan (2 bedrooms, 1 bathroom, 1 kitchen and 1 living room). For the experiments, one house was randomly assigned to be treated with the space spray formulation and the other with the surface spray formulation. Using masking tape, four 1 × 1 m squares were marked on four separate interior walls of each house (these acted as replicates for each insecticide) (Fig. S2B). Fifteen minutes prior to insecticide application, the house doors were closed and any air conditioning units were shut off. Temperature and humidity measurements were recorded during each day of experimentation. On the first day of the surface spray trials (day 0), a single application of insecticide was sprayed over the four 1 × 1 m squares in each house. Insecticide was applied from a distance of 30 cm for 10 seconds (as recommended in the label of surface sprays) (Fig. S2B). After ten minutes, four plastic cones were placed on top of each of the four squares, 25 cm inward from the square’s edges (Fig. S3). Ten mosquitoes were placed in each cone and left for 30 minutes. In total, each square had 10 mosquitoes from each strain (40 mosquitoes total). After 30 minutes, all mosquitoes were removed, placed in separate Styrofoam cups covered in mesh netting, and provided cotton soaked in 10% sucrose. Knock-down was recorded 30, 60, and 120 minutes post-exposure. Mortality was recorded after 24 hours (Fig. S2B). Cone bioassays were repeated in both houses at 2, 4 and 6 days after initial insecticide application. All cones were washed with detergent and acetone between each trial.

### kdr Genotyping

All mosquitoes from the space spray trial were genotyped to determine the presence of *kdr* mutations in both survivors and those that were knocked down. *Ae*. *aegypti* are characterized as homozygous wild type with two valine alleles at position 1016 (V/V). The SNP associated with pyrethroid resistance is an isoleucine substitution at position 1016^27,42^. For the surface spray trials, only mosquitoes tested on the day of insecticide application (day 0) and six days post-exposure were genotyped due to large sample size. Mosquito DNA was extracted using Extracta^TM^ DNA Prep for PCR (QuantaBio, Beverly, MA, USA) per the manufacturer’s instructions. DNA was analyzed by real-time PCR to isolate and amplify the allele-specific oligonucleotide sequences (5′-3′) for I1016 SNP marker. Reactions were carried out using a Bio-Rad CFX96 Real-Time System C1000 thermal cycler. PCR primers were previously described and included Val1016f, Ile1016f, and Ile1016r^42^. Eighteen µL of master mix (6 µL ddH_2_O, 10 µL iQ SYBER Green Supermix, 1 µL Val1016f, 1 µL Ile1016f, and 1 µL Ile1016r with all primers having a final concentration of 10 µM) was added to each PCR well, followed by 1 µL sample DNA. Cycling conditions included 3 minutes at 95 °C for initial DNA denaturation, followed by 35 rounds of 10 seconds at 95 °C, 10 seconds at 60 °C, and 30 seconds at 72 °C for denaturation, annealing, and extension. The reaction was then held at 95 °C for 10 seconds for the extension elongation step. Melting curves were generated by heating from 65–95 °C with 0.2 °C increments per 10 seconds, a 10 second dwell time, and plate read at each temperature. Deionized water was used as a negative control, while DNA from previously genotyped *Ae*. *aegypti* were used as positive controls. Genotype at the 1016 locus was determined through analysis of the PCR product melting curves, which were viewed using Precision Melt Analysis Software (company).

### Statistical analyses

Difference in percent mortality across insecticides and *Ae*. *aegypti* strains was determined by one- and two-way ANOVA analyses. For both the space and surface spray trials, Kaplan-Meier survival curves tested for significance using log-rank tests. Additionally, in the surface spray trials, survival analyses were performed based on daily mortality for each day post-insecticide exposure, stratified by insecticide. To further quantify the effect of each insecticide and mode of application on mosquito mortality, we performed binomial generalized linear mixed-effects models (GLMM). The main exposure variable was insecticide, followed by various predictors such as *Ae*. *aegypti* strain and I1016 allele frequency. We followed a multi-model approach in which we generated three different models including different combinations of exposure and predictor variables. Each model had experimental replicate as a random intercept. Model fit was assessed through Akaike’s information criterion (AIC). For the *kdr* data, allele frequencies were calculated as in Deming *et al*.^27^. The association between genotype and survival phenotype was calculated by Fisher’s exact tests for RxC tables^43^. All analyses were performed using SAS statistical software (Version 9.4).

### Ethics approval and consent to participate

Ethics approval was obtained from the Comisión Estatal de Bioética de Yucatán and Emory University IRB (protocol number #IRB00087229) prior to the study execution. Written, informed consent was obtained from all survey participants prior to survey administration. All research was performed in accordance with relevant guidelines/regulations.

## Electronic supplementary material

Supplementary Information

## Data Availability

The datasets used and analyzed during the current study are available from the corresponding author on request.
