# Supplementary material for: Experimental evaluation of the impact of household aerosolized insecticides on pyrethroid resistant Aedes aegypti
Source: Sci Rep. 2018 Aug 22;8:12535. doi: 10.1038/s41598-018-30968-8 (PMC6105583; doi:10.1038/s41598-018-30968-8)
Supplement: Supplementary file 1 — Supplementary Information [file 41598_2018_30968_MOESM1_ESM.docx]

**Supplementary Figures and Tables**

**Experimental evaluation of the impact of household aerosolized insecticides on pyrethroid resistant *Aedes aegypti***

Lyndsey Gray ^1^, Sergio Dzib Florez ^2^, Anuar Medina Barreiro ^2^, José Vadillo-Sánchez ^2^, Gabriela González-Olvera ^2^, Audrey Lenhart ^3^, Pablo Manrique-Saide ^2^, Gonzalo M. Vazquez-Prokopec ^4*^.

1. *Department of Epidemiology, Emory University, Atlanta, GA, USA.*
2. *Unidad Colaborativa de Bioensayos Entomológicos, Campus de Ciencias. Biológicas y Agropecuarias, Universidad Autónoma de Yucatán, Mérida, Yucatán, Mexico.*
3. *Entomology Branch, Division of Parasitic Diseases and Malaria, Center for Global Health, Centers for Disease Control and Prevention, Atlanta, GA, USA.*
4. *Department of Environmental Sciences, Emory University, Atlanta, GA, USA.*

**Figure S1.** Average 24-hour mortality quantified using the CDC bottle bioassay exposing F1 *Ae. aegypti* females from the three mosquito strains to the insecticide deltamethrin. Error bars indicate the standard deviation of the mean.


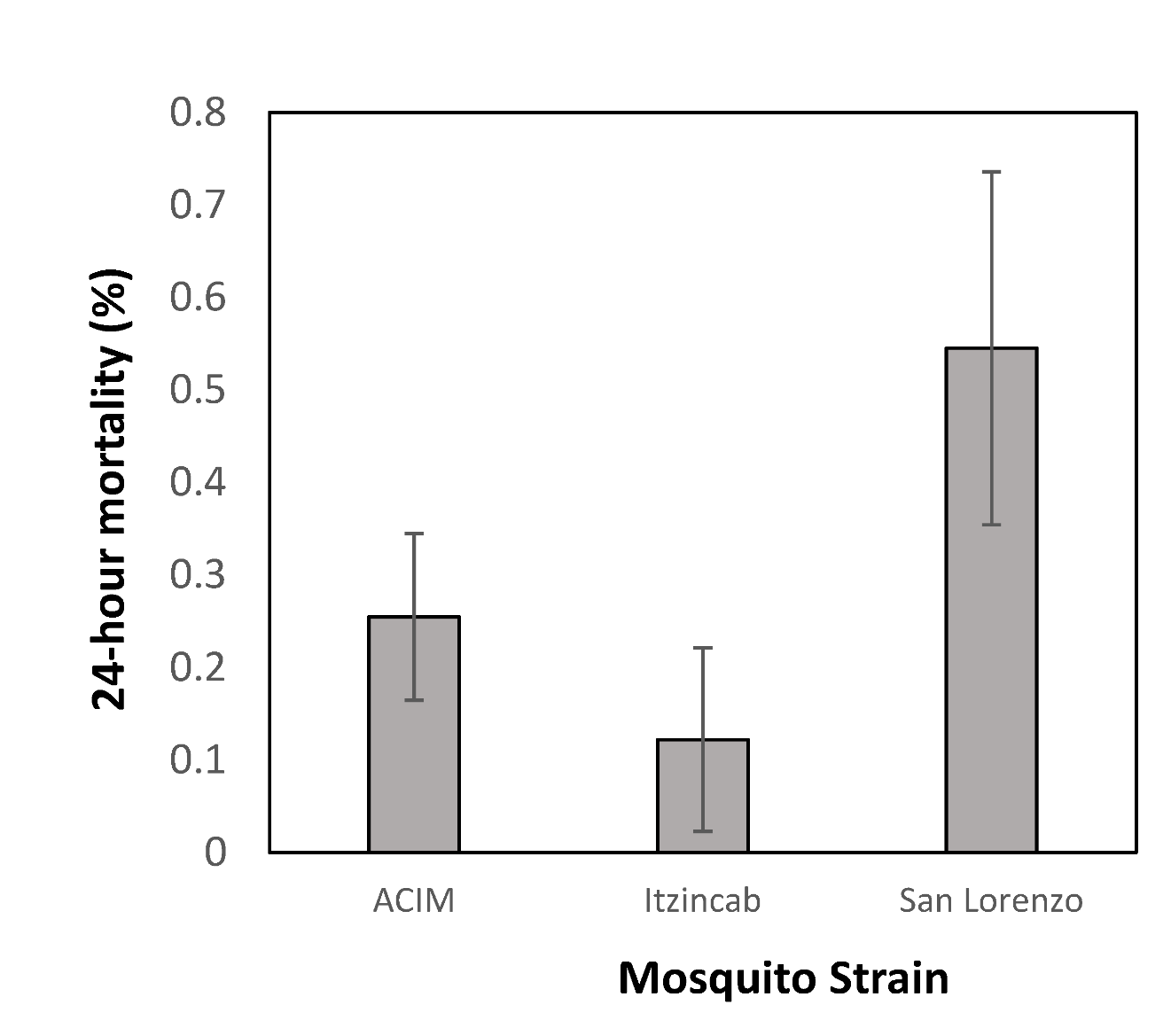


**Figure S2:** Diagram describing the design of the space (A) and surface (B) spray experiments.


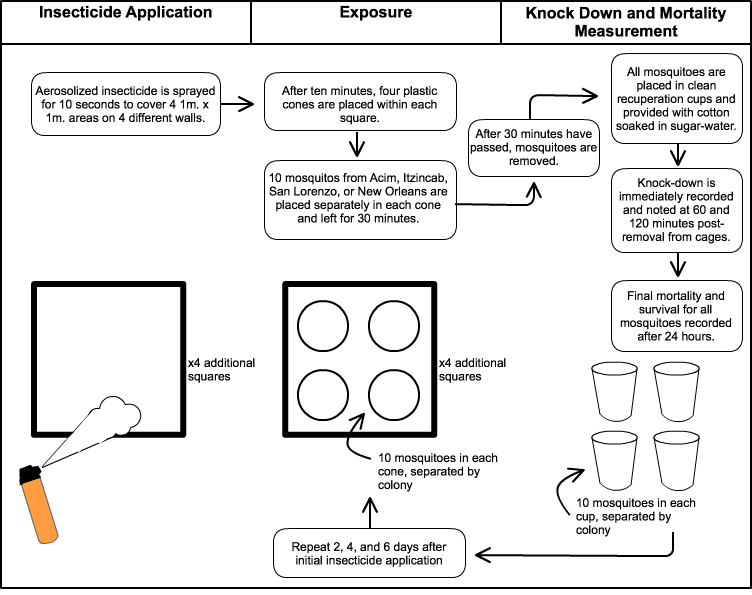

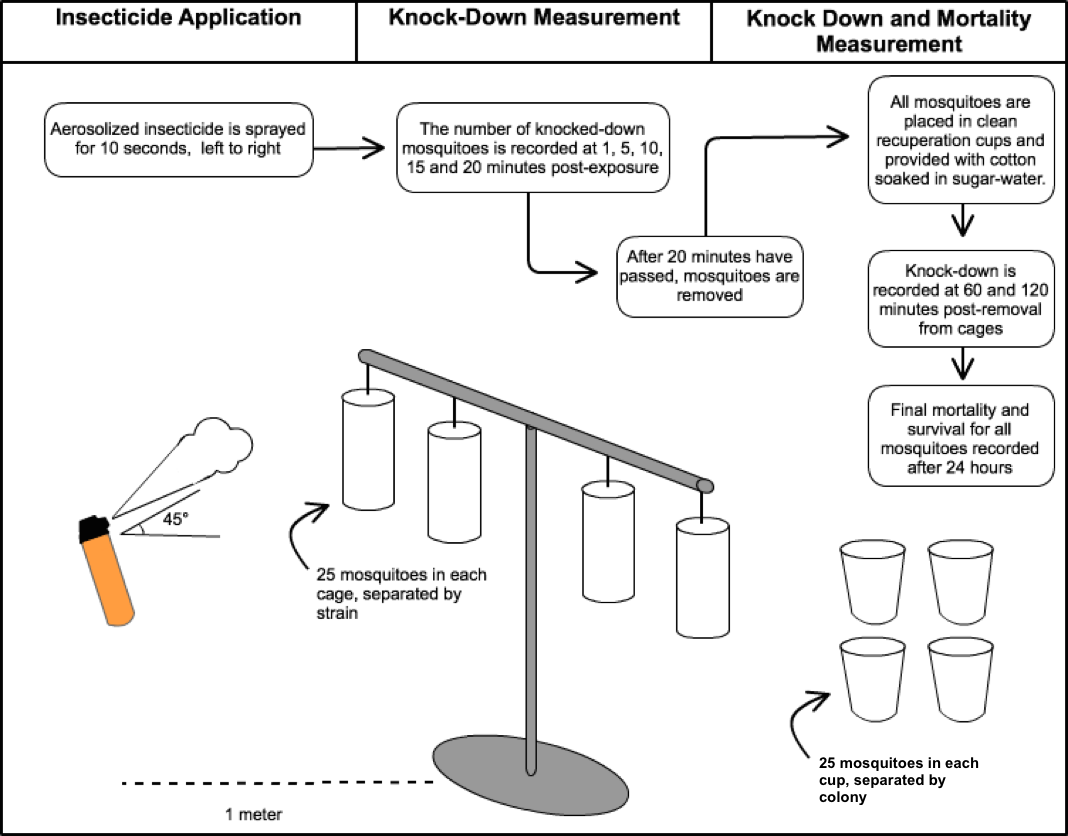


**(A)**

**(B)**

**Figure S3:** Exposure layout for mosquitoes in the surface spray trials.


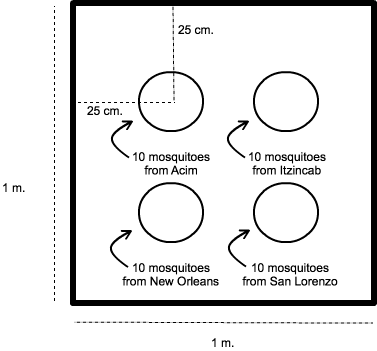


**Table S1:** Differences in knock-down time among *Ae. aegypti* exposed to aerosolized insecticide during surface spray trials.

| **Stratification Variable** | **Time Post-Exposure (Day)** | **Log-Rank Test^1^** | **P-Value** |
| --- | --- | --- | --- |
| Insecticide^2^ | 0 | 249.7 | <0.0001 |
|  | 2 | 43.9 | <0.0001 |
|  | 4 | 24.4 | <0.0001 |
|  | 6 | 31.7 | <0.0001 |
| Resistance colony^3^ | 0 | 109.3 | <0.0001 |
|  | 2 | 404.5 | <0.0001 |
|  | 4 | 173.2 | <0.0001 |
|  | 6 | 180.2 | <0.0001 |

1. Represents statistical significance among any of the four curves representing mosquito colony (New Orleans, Acim, Itzincab, and San Lorenzo) in the Kaplan-Meier analysis.
2. Comparing the two insecticide formulations (space spray vs residual spray).
3. Comparing susceptible to the aggregate of all resistant strains.
